# Cellular and Physiological Effects of Dietary Supplementation with *β*-Hydroxy-*β*-Methylbutyrate (HMB) and *β*-Alanine in Late Middle-Aged Mice

**DOI:** 10.1371/journal.pone.0150066

**Published:** 2016-03-08

**Authors:** Julian Vallejo, Madoka Spence, An-Lin Cheng, Leticia Brotto, Neile K. Edens, Sean M. Garvey, Marco Brotto

**Affiliations:** 1 Muscle Biology Research Group, School of Nursing & Health Studies, University of Missouri-Kansas City, Kansas City, Missouri, United States of America; 2 Abbott Nutrition R&D, Columbus, Ohio, United States of America; University of Debrecen, HUNGARY

## Abstract

There is growing evidence that severe decline of skeletal muscle mass and function with age may be mitigated by exercise and dietary supplementation with protein and amino acid ingredient technologies. The purposes of this study were to examine the effects of the leucine catabolite, beta-hydroxy-beta-methylbutyrate (HMB), in C_2_C_12_ myoblasts and myotubes, and to investigate the effects of dietary supplementation with HMB, the amino acid *β*-alanine and the combination thereof, on muscle contractility in a preclinical model of pre-sarcopenia. In C_2_C_12_ myotubes, HMB enhanced sarcoplasmic reticulum (SR) calcium release beyond vehicle control in the presence of all SR agonists tested (KCl, P<0.01; caffeine, P = 0.03; ionomycin, P = 0.03). HMB also improved C_2_C_12_ myoblast viability (25 μM HMB, P = 0.03) and increased proliferation (25 μM HMB, P = 0.04; 125 μM HMB, P<0.01). Furthermore, an *ex vivo* muscle contractility study was performed on EDL and soleus muscle from 19 month old, male C57BL/6nTac mice. For 8 weeks, mice were fed control AIN-93M diet, diet with HMB, diet with *β*-alanine, or diet with HMB and *β*-alanine. In *β*-alanine fed mice, EDL muscle showed a 7% increase in maximum absolute force compared to the control diet (202 ± 3vs. 188± 5 mN, P = 0.02). At submaximal frequency of stimulation (20 Hz), EDL from mice fed HMB plus *β*-alanine showed an 11% increase in absolute force (88.6 ± 2.2 vs. 79.8 ± 2.4 mN, P = 0.025) and a 13% increase in specific force (12.2 ± 0.4 vs. 10.8 ± 0.4 N/cm^2^, P = 0.021). Also in EDL muscle, *β*-alanine increased the rate of force development at all frequencies tested (P<0.025), while HMB reduced the time to reach peak contractile force (TTP), with a significant effect at 80 Hz (P = 0.0156). In soleus muscle, all experimental diets were associated with a decrease in TTP, compared to control diet. Our findings highlight beneficial effects of HMB and *β*-alanine supplementation on skeletal muscle function in aging mice.

## Introduction

Aging results in the progressive decline of many physiological processes including cognition [1], basal metabolic rate [2], cardiac output [3], pulmonary function [4] and neuromuscular activity [5]. Particularly debilitating is the severe decline in skeletal muscle mass and function with age that occurs in a subset of elders, also known as sarcopenia. Sarcopenia is a contributing factor in up to 85.6% of disability cases in elderly men and 26% in elderly women, accounting for as much as 26.2 billion dollars in health care related costs in 2000 [6]. Sarcopenia is characterized by dramatic changes in body composition, including gradual loss of skeletal muscle tissue and replacement by adipose tissue and fibrosis, resulting in decreased muscle strength and power [7,8]. Muscle weakness and frailty leads to an elevated risk of falls and injury among older persons, with increased incidence of mobility disability, loss of independence, diminished quality of life, and a host of secondary diseases [8,9]. Studies in mice, rats and humans show that these age-related changes in muscle strength are multidimensional and can only be partially explained by atrophy and loss of muscle tissue [10,11]. Additional factors contributing to muscle weakness include loss and dysmorphology of motor units [12], reduced myofiber cross sectional area, increased non-contractile tissue, reduced satellite cell activity [13] and altered myocellular calcium homeostasis [14–18]. These size-independent factors highlight the importance of maintaining muscle quality (strength per unit of muscle mass) during aging, over muscle quantity alone.

Resistance exercise is an effective treatment for countering muscle mass and strength losses in elderly and sarcopenic individuals [8,19–22]. However, regular exercise is not always feasible for elderly adults, who may be more susceptible to injury and prolonged recovery, and especially those with mobility disability or other chronic diseases [8,23]. Additionally, pharmacological and dietary interventions have been implemented to counter muscle loss with aging [8,24,25]. There is a growing body of evidence showing that dietary supplementation with protein [26], branched-chain amino acids (BCAAs) [27], and even amino acid metabolites [28] can mitigate sarcopenia when taken alone or in combination with resistance training. However, mechanisms of action and synergies for some of these interventions are not well understood.

One promising dietary intervention is beta-hydroxy-beta-methylbutyrate (HMB), a metabolite of leucine, an essential BCAA. It is estimated that 5% of leucine metabolism results in production of HMB *in vivo* [29], while the majority of leucine catabolites are shunted toward production of HMG-CoA, which is either converted to acetyl-CoA for tricarboxylic acid cycling or possibly utilized for cholesterol biosynthesis [30]. In rats, Pinheiro et al demonstrated that HMB supplementation increased *in situ* gastrocnemius muscle tetanic force production and resistance to fatigue [31]. Also in rats, Alway et al demonstrated that HMB enhanced satellite cell activity and proliferation following muscle unloading [32]. Furthermore, HMB treatment has been reported to act on markers of muscle protein turnover by both decreasing muscle catabolism through modulation of the ubiquitin-proteasome proteolytic pathway and stimulating muscle protein synthesis via mTOR/p70S6k, IGF, MAPK/ERK or PI3/Akt pathways [32–38]. In myoblast culture, HMB has been shown to induce expression of the proliferation marker MyoD and the differentiation markers MEF2 and myogenin [34].

HMB initially gained traction as a dietary aid through sports performance studies in physically active male and female young adults [39]. HMB has since been demonstrated to promote muscle mass, strength, power, or physical performance or reduce skeletal muscle atrophy in elderly subjects [40–44]. HMB supplementation also prevented the loss of lean mass in a human study of 10 days of bed rest [45], and has shown similar efficacy in experimental models of cancer cachexia [37,46–48]. On the other hand, several studies, namely in strength-trained athletes, do not support these results and may reflect differences in dose and duration of HMB supplementation or total protein intake [49–51].

Another hopeful dietary ingredient to counteract age-related loss of strength is beta-alanine (*β*-alanine)–a non-proteinogenic amino acid most abundant in the central nervous system and skeletal muscle. In muscle, *β*-alanine and _L_-histidine combine to form the myoprotective dipeptide carnosine [52,53]. As a dietary supplement, *β*-alanine has been shown to enhance physical exercise capacity and the threshold for fatigue in both young [54] and elderly humans [55,56], possibly via carnosine’s enhanced intramyofibrillar H^+^ buffering during exercise-induced metabolic acidosis. A recent study in human skinned muscle myofibers [57], as well as studies in rat [58] and other non-mammalian species [59], provide compelling evidence that carnosine enhances the intrinsic sensitivity of the contractile apparatus to calcium in a dose-dependent manner. Additionally, elevated intramuscular *β*-alanine and carnosine levels, achieved through dietary supplementation in mice, correlated with both increased resistance to fatigue in soleus muscle and enhanced excitation-contraction (EC) coupling in the extensor digitorum longus (EDL) muscle through a leftward shift (10–31% higher forces) of the force-frequency curve [60]. In some studies, though, *β*-alanine supplementation did not show an impact on muscle carnosine concentrations or physical exercise capacity [61–63].

The primary objective of the present study was to test for an interaction between dietary HMB and *β*-alanine supplementation on muscle strength, muscle quality, and resistance to and recovery from fatiguing stimulations in a mouse model of pre-sarcopenia. We predicted that dietary intervention with HMB would increase muscle force through improved myofibrillar protein accretion, while *β*-alanine would improve muscle quality and resistance to and recovery from fatigue via enhanced E-C coupling. We also hypothesized an additive benefit of HMB and *β*-alanine co-supplementation, in particular with regard to muscle strength and muscle quality. We also tested whether HMB treatment had an effect on proliferation and intracellular calcium handling in differentiated murine C_2_C_12_ myotubes.

## Materials and Methods

### Cell viability and proliferation

To assess the effect of HMB on C_2_C_12_ myoblast viability, the Trypan blue exclusion assay was performed. C_2_C_12_ myoblasts were plated in non-coated 6-well plates at a density of 1600 cells per cm^2^ and synchronized at G_0_/G_1_ for 24 hours under conditions of serum deprivation [1% fetal bovine serum (FBS) in DMEM]. Media was then switched to low serum (3% FBS) or normal serum (10% FBS) proliferation media containing either 25 or 125 μM HMB free acid or an equivalent volume of PBS (vehicle control). After 48 hours and 72 hours of proliferation, cells were trypsinized, stained with Trypan blue (catalog #1691049, MP Biomedicals, Santa Ana, CA, USA) and counted manually using a hemocytometer (catalog #1492, Hausser Scientific, Horsham, PA, USA). Cell viability was calculated as the percent of unstained cells [64,65]. The experiment was repeated 3 times. To determine the effect of HMB on C_2_C_12_ myoblast proliferative capacity, the Scepter™ automated cell counter (EMD Millipore, Billerica, MA, USA) was used. C_2_C_12_ myoblasts were plated in non-coated 24-well plates at a density of 1600 cells per cm^2^ and synchronized at G_0_/G_1_ for 24 hours under conditions of serum deprivation (1% FBS in DMEM). Media was then switched to proliferation media (10% FBS in DMEM) supplemented with either 25 μM or 125 μM HMB or an equivalent volume of PBS. At 48 hours of proliferation, cells were trypsinized and counted using the Scepter™ assay. To obtain the most accurate cell count, the results were gated to exclude cellular debris [66]. Free acid HMB (catalog #55453, Sigma-Aldrich, St. Louis, MO, USA) was used in all cell culture studies, whereas calcium HMB monohydrate was used in the in vivo animal study. Data is presented as mean ± SEM.

### Fura-2 monitoring of intracellular Ca2^+^

For quantitative measurements of intracellular [Ca^2+^] in cultured myoblasts, we employed methods previously developed and described by the Brotto Laboratory [64,67]. C_2_C_12_ myoblasts were plated on 25 mm^2^ glass cover slips at a density of 8000 cells per cm^2^ and allowed to differentiate into myotubes by treating the cells for 7 days with differentiation media (2.5% horse serum in DMEM) containing either 25 μM or125 μM HMB or vehicle control. After 7 days of differentiation, the myotubes were loaded with 4 μM Fura-2-AM for 40 minutes, then washed, at which time Fura-2 was allowed to de-esterify. As myotube motion artifacts are associated with intracellular Ca^2+^ release, 10 μM N-benzyl-p-toluene sulfonamide (Sigma-Aldrich), a specific myosin II inhibitor, was applied for 20 min. The cells were then mounted on an inverted microscope equipped with a dual-wavelength (excitation at 340 nm and 380 nm) spectrofluorometer (Photon Technology International, Birmingham, NJ, USA) which was used to determine the change in magnitude of intracellular Ca^2+^ transients in response to KCl, caffeine, and ionomycin. Specifically, cells were perfused for 2 min each with 80 mM KCl, followed by 20 mM caffeine and finally 10 μM ionomycin. The cells were washed with physiological buffer and allowed to recover to normal resting calcium levels between each chemical stimulation. The use of KCl, caffeine and ionomycin provide information on depolarization-induced calcium release, calcium-induced calcium release and total sarcoplasmic reticulum calcium storage [68], respectively. Data is presented as mean ± SEM.

### Animals

All experimental procedures were approved by the Institutional Animal Care & Use Committees at the University of Missouri Kansas City (Kansas City, MO, USA). Male virgin C57BL/6NTac mice (8 mice each of ages 14, 14.5, 15, 15.5, 16 and 16.5 months; 48 mice total) were purchased from Taconic Farms, Inc. (Germantown, NY, USA) and housed individually with *ad libitum* access to food and water and a 12 h/day light cycle for the duration of the study. All Mice were fed normal chow while acclimating to the facility and aged to 17 months, followed by 2 weeks of acclimation to control purified diet (AIN-93M diet, catalog TD.94048, Harlan Laboratories, Inc., Indianapolis, IN, USA). At this point, the mice were distributed across 4 experimental groups fed control purified diet, purified diet containing calcium HMB monohydrate (514 mg/kg bw, hereafter referred to as HMB), purified diet containing *β*-alanine (411 mg/kg bw), or the combination of HMB and *β*-alanine (HMB: 514 mg/kg; *β*-alanine: 411 mg/kg bw). HMB (LOT# DO42364, Lonza Group Ltd., Basel, Switzerland) and *β*-alanine (LOT# 109782, Yuki Gosei Kogyo Co., Ltd., Tokyo, Japan) were provided by Abbott Nutrition (Columbus, OH, USA). Mice were fed experimental diets for 8 weeks. Food intake and body weight were recorded daily during the first week of dietary supplementation (to test for need to pair feed), and then on a weekly basis thereafter. Pair feeding was not necessary, and no mice died during the course of the study. Following the dietary intervention period, mice were sacrificed by cervical dislocation and the EDL and soleus muscles were carefully removed for contractility analysis, as previously reported [69]. All mice were 19 months old at sacrifice.

### *Ex vivo* muscle contractility

Dissected intact EDL and soleus muscles were immediately placed in a dish containing a HEPES Ringer solution (143 mM NaCl; 5 mM KCl; 1.8 mM MgCl_2_; 10 mM HEPES; 2.5 mM CaCl_2_; pH 7.40) with 10 mM glucose. This solution was continuously aerated with 100% O_2_. This experimental bathing solution was used to tightly control pH, and our results show that maximal tetanic forces are within the range of normalized forces reported for bathing solutions that optionally use bicarbonate and phosphate and a mixture of CO_2_ and O_2_ [69]. These results are also in agreement with our previous studies on the effects of hypoxia and hypoxia-induced fatigue in diaphragm muscles from mice [70]. EDL and soleus muscles were mounted vertically between two stimulating platinum electrodes (Monrovia, CA, USA) and immersed in a 25 ml bathing chamber containing the Ringer solution. Via the tendons, the muscles were suspended from adjustable isometric force transducers above the chambers and secured to the base of the tissue support within the chambers. The analog output of the force transducer was digitized, stored and analyzed with PowerLab^®^ Software (ADInstruments Inc., Colorado Springs, CO, USA). For each muscle the stimulatory voltage was provided by a S8800 dual pulse digital stimulator (Grass Products, West Warwick, RI, USA) (pulse duration, 1 ms; train duration, 500 ms). Optimal muscle length (*L*_0_) was first determined for each muscle by lengthening the muscle until tetanic stimulations of 100 Hz at an interval of 1 min produced maximal force. *Equilibration*: Next the intact EDL and soleus muscles were allowed a 30 minute equilibration period during which time they were stimulated with pairs of alternating high (80 Hz) and low (20 Hz) frequency pulse-trains administered with a periodicity of 1 min to mimic normal muscle activity. Utilization of the proposed paradigm of stimulation helps with the study of the relative contributions of the contractile proteins (80Hz) and the sarcoplasmic reticulum (20Hz) to contractile function [69,71]. *Force-Frequency Relationship*: Following equilibration, the EDL and soleus muscles were subjected to stimulation with frequencies ranging from 1–130 Hz with a periodicity of 1 min to generate the force vs. frequency (FF) relationship. The peak force of each contraction at the different stimulation frequencies were used to plot the force-frequency relationship. *Fatigue*: Next, to induce fatigue, the EDL and soleus muscles were stimulated with pairs of alternating high (80 Hz) and low (20 Hz) frequency pulse trains with a periodicity of 1 sec for 5 min. The extent of fatigue was determined as the percent of force remaining after the 5 min fatiguing protocol (relative to force at 80Hz and 20Hz just prior to fatiguing stimulations). *Recovery from fatigue*: Immediately following the fatiguing protocol, the EDL and soleus muscles were allowed 30 min recovery period during which time alternating stimulatory trains of high (80 Hz) and low (20 Hz) frequency were applied with a periodicity of 1 min. The muscles were then allowed a second 30 min recovery period with 5mM caffeine added to the chamber bath to gauge overall EC coupling during the recovery period [14,68,69]. Although caffeine may exert its effects via a combination of sites within the muscle (Ryr Ca^2+^ leak, SR Ca^2+^ pump rate, contractile machinery Ca^2+^ sensitivity), the level of muscle force recovery observed in the presence of caffeine indicates that the effects are likely due to Ca^2+^ availability during EC coupling [72,73]. *Force data*: Muscle force is reported as absolute force (mN) and force normalized to muscle physiological cross sectional area (N/cm^2^) as previously reported by our group [69,74,75]. *Slope data*: The slope of the rising edge of muscle contractions was measured 0–31 ms after the start of the peak. *Time to peak data*: Calculated as the time (ms) it took to reach peak force from the start of a muscle contraction. *Tau data*: Tau was calculated from 90%-0% of peak height during the relaxation phase of muscle contractions. All data is presented as mean ± SEM.

### Statistical Analysis

#### *In vitro* cell studies

*In vitro* cell studies were statistically analyzed using one-way analysis of variance (ANOVA) looking at the main effect with P<0.05 considered significant.

#### Contractility

Data points that were three standard deviations away from the mean were considered outliers and removed from the data set. Kolmogorov-Smirnov test was conducted to test for the normality assumption of the outcomes variables. All variables passed the normality test under the significance level of 0.05. A Mixed model approach was conducted to test for the treatment effect while accounting for the repeated measures on the same individual. Pairwise contrasts were estimated and tested for significance between different treatments. Due to the multiple comparisons of the contrasts, a more strict alpha level of 0.025 was used to establish the statistical significance.

## Results

### *In vitro* cell studies

**Fig 1:**
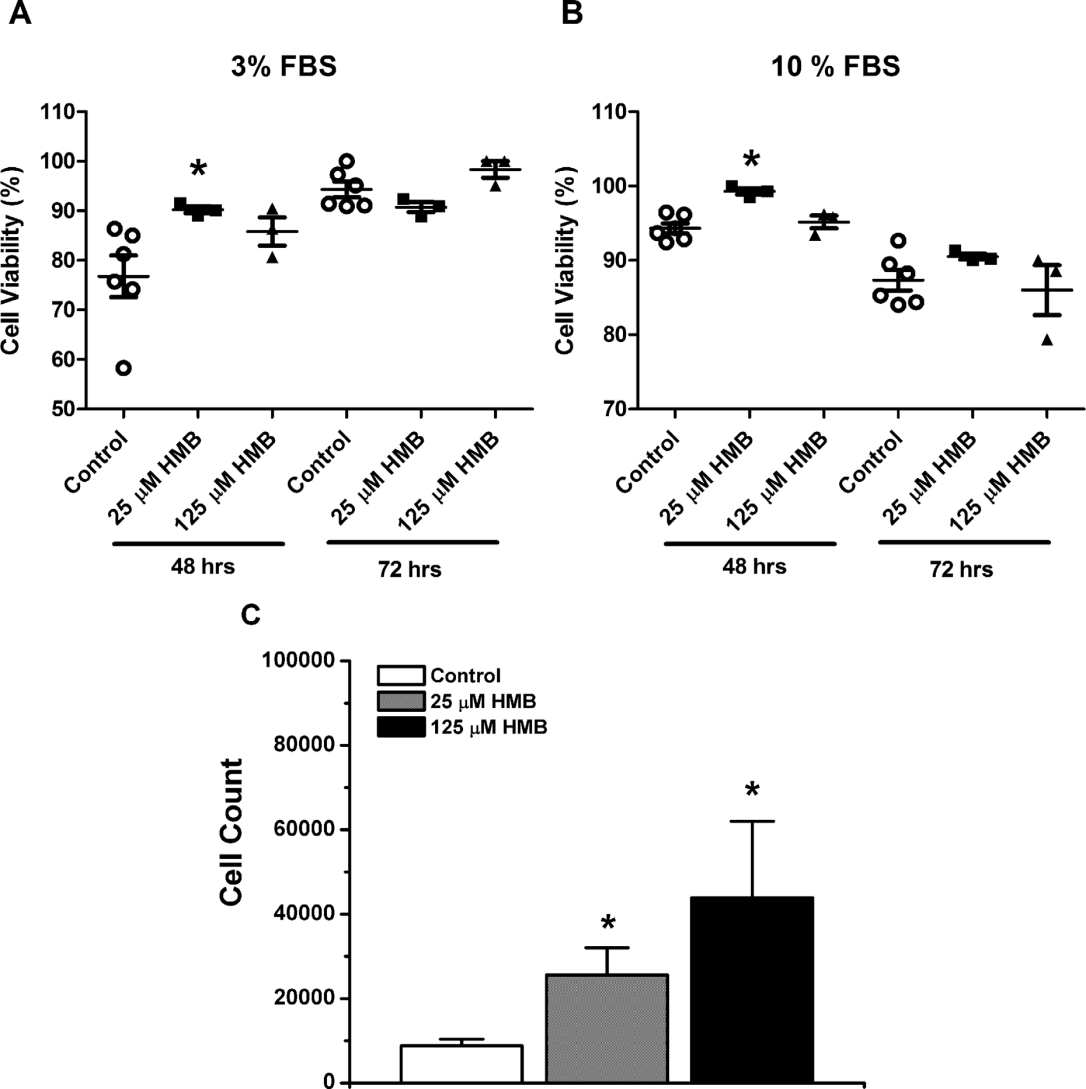
HMB enhances C_2_C_12_ myoblasts cell viability and proliferation. C_2_C_12_ myoblasts were treated with either 25 μM or 125 μM free acid HMB or vehicle control. Cell viability was assessed at 48 and 72 hours proliferation using the Trypan Blue exclusion assay. Experiments were performed under conditions of both low serum (A) and normal serum (B), (Control, n = 6; 25 μM HMB, n = 3; 125 μM HMB, n = 3, * denotes significant difference compared to control: 3% FBS, P = 0.044; 10% FBS, P = 0.031, One-way ANOVA). C) C_2_C_12_ myoblasts were treated with 25 μM or125 μM HMB or vehicle control for 48 hours in proliferation media (10% FBS) at which point total viable cells were counted while gating out cellular debris with the Scepter™ Automated Cell Counter (Control, n = 6; 25 μM HMB, n = 3; 125μM HMB, n = 3,* denotes significance compared to control: 25 μM HMB, P<0.042; 125 μM HMB, P<0.01, One-way ANOVA).

The viability of C_2_C_12_ myoblasts, under conditions of low serum/cell starvation and normal serum, was significantly enhanced after 48 hours of proliferation in the presence of 25 μM HMB compared to control (Fig 1A and 1B). We did not observe any significant increases in cell viability beyond 48 hours of proliferation under these conditions. HBM treatment at 125 μM had no effect on cell viability. Both doses of HMB (25 μM and 125 μM) enhanced C_2_C_12_ myoblast proliferation after 48 hours in media containing 10% serum (Fig 1C). These results were obtained using an automated cell counter.

**Fig 2:**
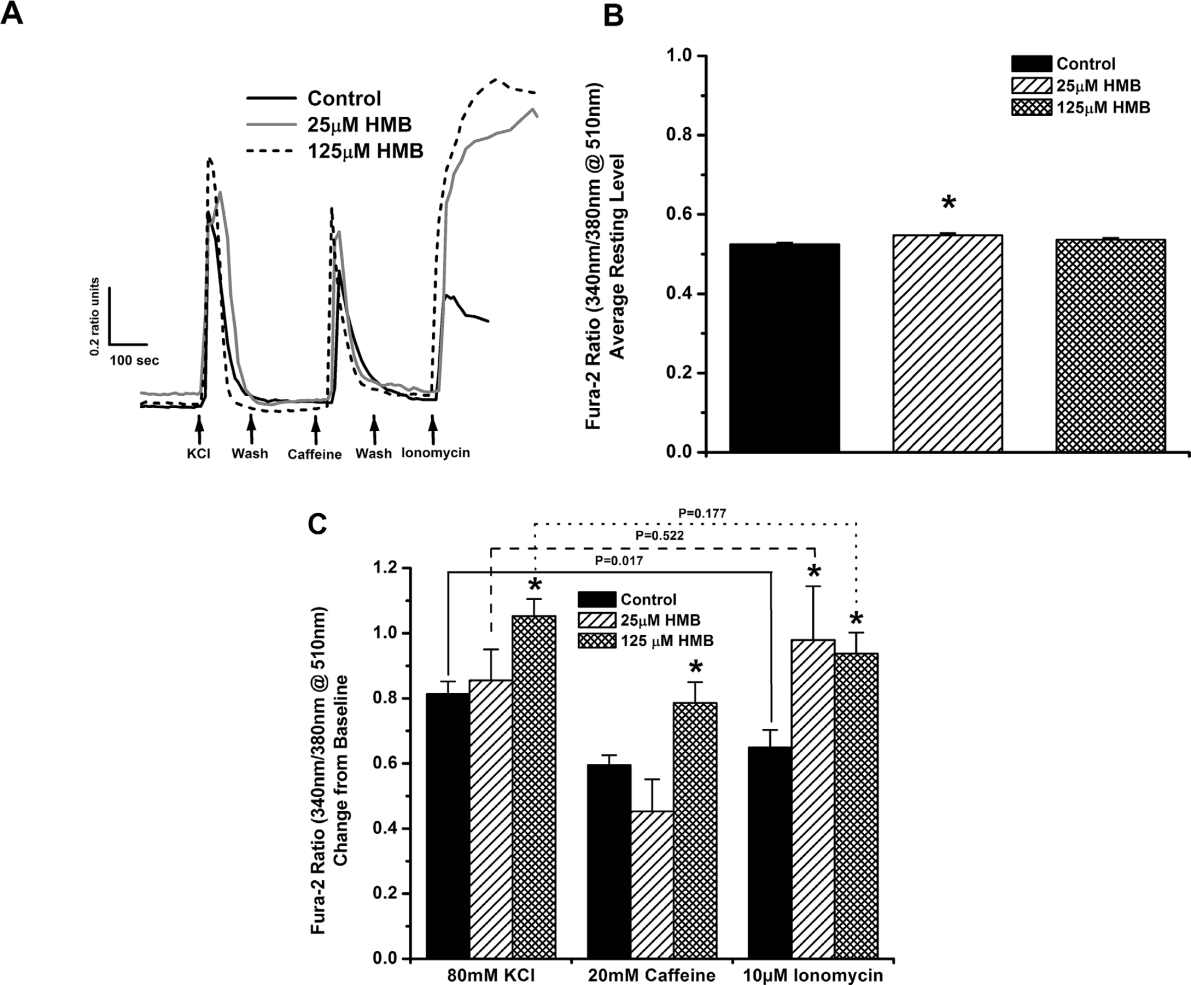
HMB increases depolarization-induced calcium release, caffeine induced calcium release from the SR, and total calcium storage of C_2_C_12_ myotubes. Calcium responses from mature C_2_C_12_ myotubes differentiated for 7 days in the presence of 25 μM or 125 μM HMB or vehicle were measured after perfusion with 80 mM KCl, 20 mM caffeine and 10 μM ionomycin. A) Representative calcium responses from control, 25 μM HMB and 125 μM HMB treated C_2_C_12_ myotubes measured with Fura-2. Arrows indicate times of perfusion of the cells with KCl, caffeine and ionomycin as well as washout sections between chemical stimulations. B) Average resting levels of cytosolic calcium in control, 25 μM HMB and 125 μM HMB treated C_2_C_12_ myotubes (n = 15–20 cells, * denotes P<0.01 compared to control, one-way ANOVA). C) Peak change in the Fura 2 ratio from baseline in control, 25 μM HMB and 125 μM HMB treated C_2_C_12_ myotubes in response to KCl, caffeine, and ionomycin treatments. (n = 15–20 cells, *denotes significance compared to control: KCl, P<0.01; Caffeine, P = 0.032; Ionomycin, P = 0.03, One-way ANOVA). HMB-treated myotubes, but not control, were able to maintain robust release of calcium between the first and last chemical treatment as the change in Fura 2 ratio (C) values were significantly decreased between 80 mM KCl and 10 μM ionomycin treatment in control myotubes but not in 25and 125 μM HMB treated cells (Fura-2 ratio of 80 mM KCl treatment compared to 10 μM ionomycin in the same group: Δ Fura-2 Ratio (C): 25 μM HMB-P = 0.522, 125 μM HMB: P = 0.177, Control-P = 0.017)

Next, we monitored intracellular calcium levels ([Ca^+2^]_i_) under resting conditions, followed by consecutive sarcoplasmic reticulum (SR) Ca^+2^ release stimulation with 80 mM KCl, 20 mM caffeine, and 10 μM ionomycin in differentiated C_2_C_12_ myoblasts (Fig 2A). Myoblasts were differentiated for 7 days into multinucleate myotubes in the presence of both 25 μM and 125 μM HMB. Myotubes differentiated with 25 μM HMB displayed a slight but significant increase in the resting level of cytosolic calcium compared to control myotubes (P<0.01, one-way ANOVA) (Fig 2B). These myotubes also released significantly more calcium in response to ionomycin (P = 0.026, one-way ANOVA) but displayed similar responses to KCl and caffeine when compared to control myotubes (Fig 2C). In addition, 125 μM HMB was able to induce an overall significantly larger SR Ca^+2^ release for all three agonists, described as the change from baseline of the Fura-2 ratio compared to control (KCl, P<0.01; Caffeine, P = 0.032; Ionomycin, P = 0.03, one-way ANOVA) (Fig 2C). It is also noticiable that under control conditions there is a significant decrease in calcium release from the consecutive challlenges of the cells to the three calcium releasing agents (Fig 2C, bracketed comparisons). In contrast, both 25 μM 125 μM HMB treatment spared the cells from this decline in calcium release (Fig 2C, bracketed comparisons).

### *Ex vivo* contractility

**Fig 3:**
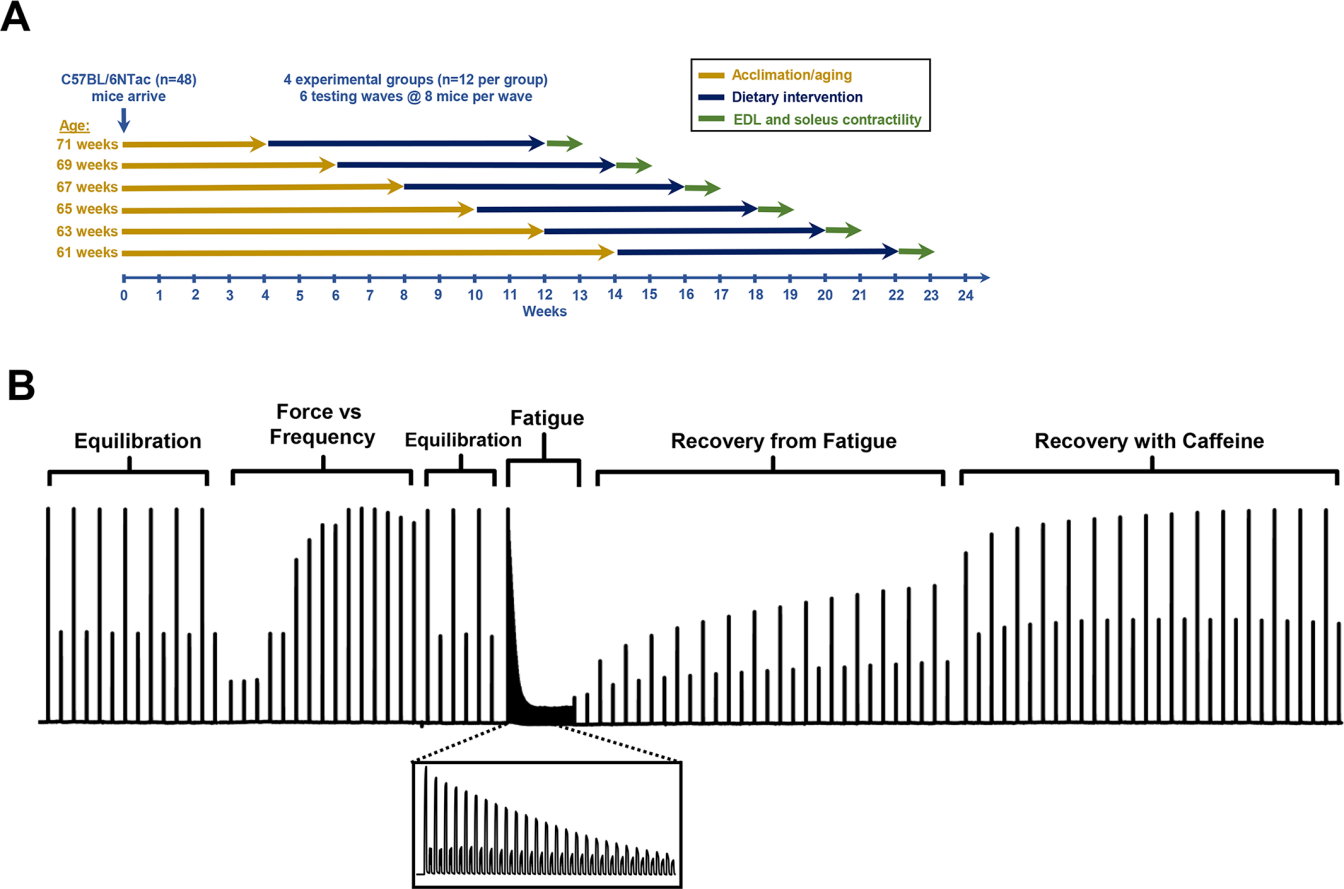
Summary of *in vivo* study timeline and muscle contractility assay. A) Schematic representation of the dietary intervention study performed on 48 C57BL/6nTac male mice. All mice were 19 months old at the time of sacrifice for contraction studies. B) Representative tracing of force data from the *ex vivo* contractility assay obtained from one muscle (*X-axis*: Time; *Y-axis*: Force). Equilibration, fatigue, and recovery from fatigue protocols are performed using alternating high (80 Hz) and low (20 Hz) frequencies of stimulation. *Inset*: Magnified view of fatiguing stimulations using alternating high (80 Hz) and low (20 Hz) stimulation frequencies.

A total of 48 late middle-aged male mice were fed control purified diet, diet plus HMB, diet plus *β*-alanine, or diet plus the combination of HMB and *β*-alanine. Fig 3A shows a timeline for the dietary intervention study performed on these mice. During the 8 weeks of dietary supplementation, there were no significant differences in body weight or food intake across the 4 experimental groups. The range in average body weight across all four experimental groups was 42.8–44.1 g. At the end of study, muscle wet weights and optimal lengths showed no differences across the four experimental groups (Table 1).

**Table 1:** EDL and soleus muscle optimal length and mass.

**EDL**	**Avg. muscle optimal length (cm)**	**SEM**	**N**	**P-value**	**Avg. muscle mass (mg)**	**SEM**	**N**	**P-value**
**Control**	1.367	0.0175	24		10.8	0.187	24	
**CaHMB**	1.392	0.0133	24	0.688	11.1	0.176	24	0.706
***β*-alanine**	1.390	0.0181	24	0.743	11.2	0.130	24	0.326
**HMB + *β*-alanine**	1.369	0.0147	24	1.000	10.6	0.193	24	0.806
**Soleus**	**Avg. muscle optimal length (cm)**	**SEM**	**N**	**P-value**	**Avg. muscle mass (mg)**	**SEM**	**N**	**P-value**
**Control**	1.210	0.0168	24		11.3	0.249	24	
**CaHMB**	1.194	0.0184	24	0.890	10.8	0.327	24	0.219
***β*-alanine**	1.188	0.0148	24	0.757	10.9	0.279	24	0.716
**HMB + *β*-alanine**	1.194	0.0154	24	0.890	10.7	0.276	24	0.837

**Fig 4:**
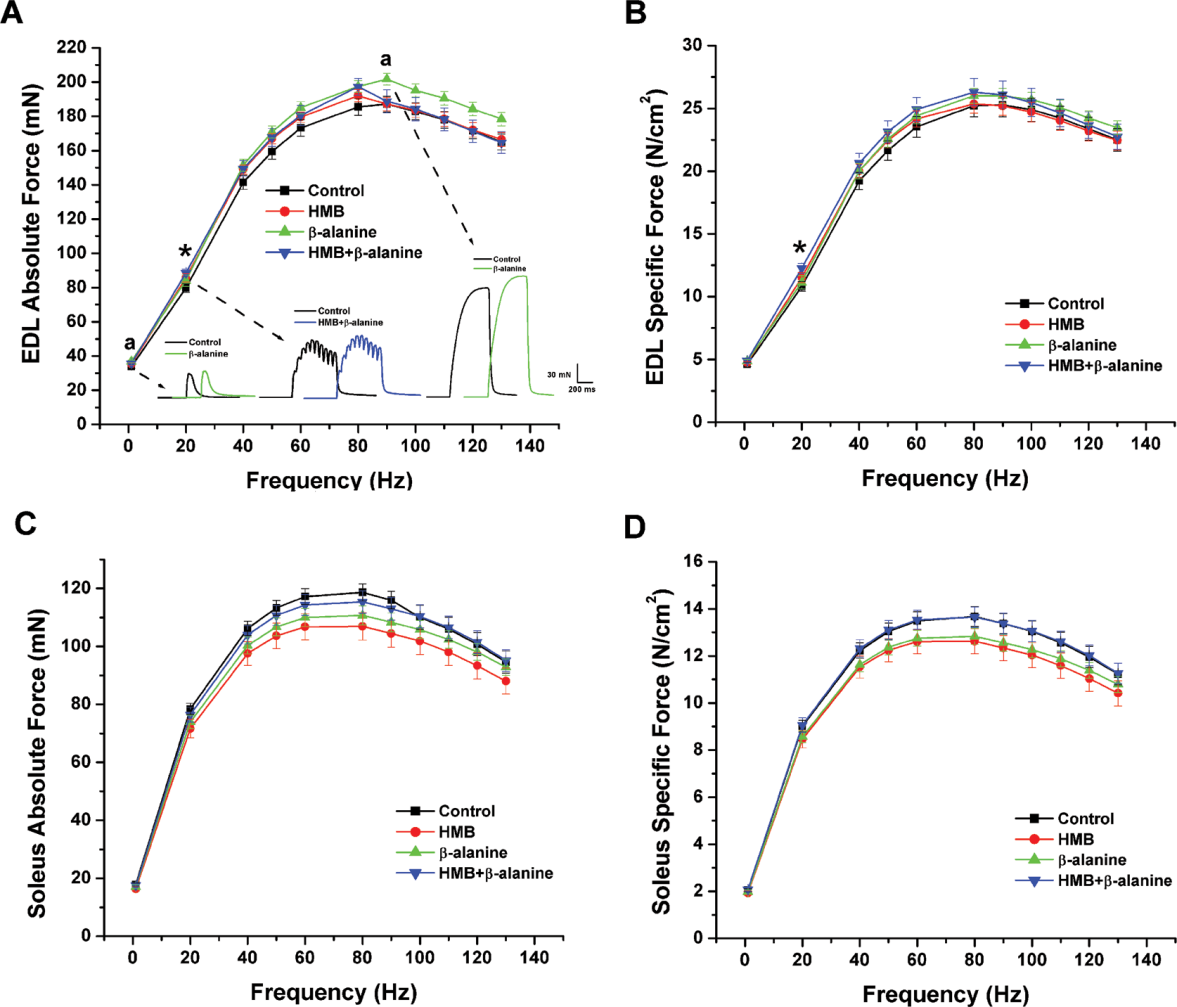
EDL and soleus muscle force vs. frequency relationships. Muscle contraction was stimulated with increasing frequencies from 1–130 Hz to determine the force-frequency relationship. A) EDL muscle absolute force. *Inset*: Raw data traces of 1 Hz, 20 Hz and maximal tetanic contractions (* denotes P<0.025, HMB + *β*-alanine compared to control diet; ^**a**^ denotes P<0.025, *β*-alanine compared to control diet). B) EDL muscle specific force (* denotes P<0.025, HMB + *β*-alanine compared to control diet). C) Soleus muscle absolute force. D) Soleus muscle specific force. (EDL: Control-n = 24 muscles, HMB-n = 23 muscles, *β*-alanine-n = 24 muscles, HMB + *β*-alanine-n = 24 muscles. SOL: Control-n = 23 muscles, HMB-n = 24 muscles, *β*-alanine-n = 24 muscles, HMB + *β*-alanine-n = 24 muscles).

Included in the contractility analysis were 12 animals per group, with 24 EDL muscles and 24 soleus (SOL) muscles analyzed per group (muscles from both hindlimbs per mouse were tested). Representative data from a contractility experiment are shown in Fig 3B. The force vs. frequency relationship for the EDL muscles from the *β*-alanine group revealed a 9% higher absolute twitch force at 1 Hz (P *=* 0.009), compared to control muscles (Fig 4A). Dietary HMB and *β*-alanine co-supplementation increased both absolute and specific submaximal (20 Hz stimulation frequency) force generation in EDL muscle by 11.0% (88.6 mN ± 2.2 mN vs. 79.8 mN ± 2.4 mN, P = 0.025) and 13.4% (12.2 N/cm2 ± 0.4 N/cm2 vs. 10.8 N/cm2 ± 0.4 N/cm2, P = 0.021), respectively, compared to control (Fig 4A and 4B). In addition, maximal tetanic force in the EDL muscle was 7.3% higher (202.0 mN ± 3.4 mN vs. 188.2 mN ± 4.6 mN, P = 0.023) in mice supplemented with *β*-alanine alone (Fig 4A). In the soleus muscle, however, no significant change in either absolute or specific force generation was detected (Fig 4C and 4D).

**Fig 5:**
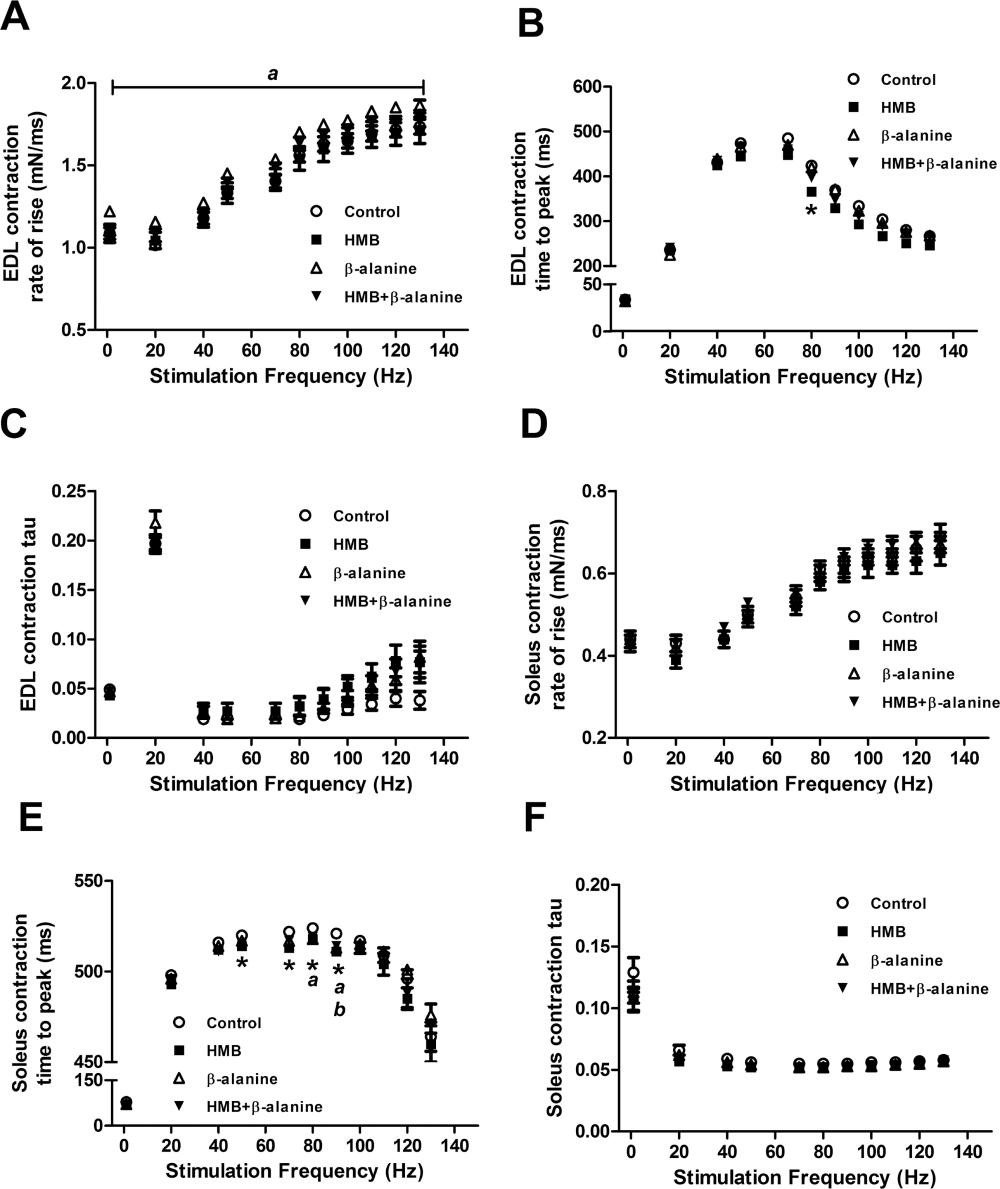
Kinetic properties of individual contractions from EDL and soleus muscles. A) Rate of force generation of individual contractions from EDL muscle at the stimulatory frequencies ranging from 1–130 Hz (^**a**^ denotes significant difference in *β*-alanine group compared to control diet. The bracket indicates significance at all stimulatory frequencies tested). B) The time to reach peak contractile force in contractions from EDL muscle stimulated with the frequencies of 1–130 Hz (* denotes significant difference in HMB diet compared to control diet). C) The time constant (tau) in the decaying exponential fit to the tail of the contractions of EDL muscle stimulated at 1–130 Hz. D) Rate of force generation of individual contractions from soleus muscle at the stimulatory frequencies ranging from 1–130 Hz (^**a**^ denotes significant difference in *β*-alanine group compared to control. E) The time to reach peak contractile force in contractions from soleus muscle stimulated with the frequencies of 1–130 Hz (* denotes significant difference in HMB diet compared to control diet, ^**a**^ denotes significant difference in *β*-alanine group compared to control, ^**b**^ denotes significant difference in HMB + *β*-alanine group compared to control). F) The time constant (tau) in the decaying exponential fit to the tail of the contractions of soleus muscle stimulated at 1–130 Hz. (EDL: Control-n = 24 muscles, HMB-n = 23 muscles, *β*-alanine-n = 24 muscles, HMB + *β*-alanine-n = 24 muscles. SOL: Control-n = 24 muscles, HMB-n = 24 muscles, *β*-alanine-n = 24 muscles, HMB + *β*-alanine-n = 24 muscles).

We also analyzed kinetic properties of individual muscle contractions from the force-frequency relationship, including the rate of rise of the muscle contraction and the time required to reach peak contractile force. The rate, or slope, of force development in the EDL muscles from the *β*-alanine group was significantly increased at all stimulatory frequencies tested, compared to control diet (1–130Hz, P<0.025) (Fig 5A). Twitch (1 Hz) and maximal force (80 Hz) contractions, which displayed significantly increased force within the *β*-alanine group, showed a 12% and 4% increase in the rate of rise, respectively (Control vs *β*-alanine-twitch: 1.08 ± 0.03 mN/ms vs. 1.22 ± 0.02 mN/ms, P = 0.0022. Control vs *β*-alanine-80 Hz: 1.56 ± 0.04 vs. 1.70 ± 0.03, P = 0.0072) (Fig 5A). EDL muscles from the HMB group required 58 milliseconds less time to reach peak maximum contractile force at 80 Hz, compared to control (366.0 ± 20.0 ms vs. 423.9 ± 10.9 ms, P = 0.0156) (Fig 5B). In soleus, no significant differences were detected in the slope of contractions between any of the experimental groups (Fig 5D). However, the time required to produce peak contractile force was reduced in all intervention groups (Fig 5E). HMB alone reduced the time to peak at the stimulatory frequencies of 50–90 Hz (Control vs HMB-50 Hz: 520 ± 2 ms vs. 514 ± 1 ms, P = 0.0238; 70 Hz: 522 ± 2 ms vs. 513 ± 2 ms, P = 0.0009; 80 Hz: 524 ± 2 ms vs. 518 ± 1 ms, P = 0.0246; 90 Hz: 521 ± 2 ms vs. 511 ± 1 ms, P = 0.0004) while contractions from the *β*-alanine and HMB + *β*-alanine groups peaked more quickly at 80–90 Hz (Control vs *β*-alanine -80 Hz: 524 ± 2 ms vs. 518 ± 2 ms, P = 0.0108; 90 Hz: 521 ± 2 ms vs. 513 ± 2 ms, P = 0.0029) and 90 Hz alone (Control vs HMB+*β*-alanine -90 Hz: 521 ± 2 vs. 513 ± 2 ms, P = 0.0140), respectively (Fig 5E). There were no significant differences detected in the relaxation properties (tau) of either EDL (Fig 5C) or soleus muscles across experimental groups (Fig 5F).

**Fig 6:**
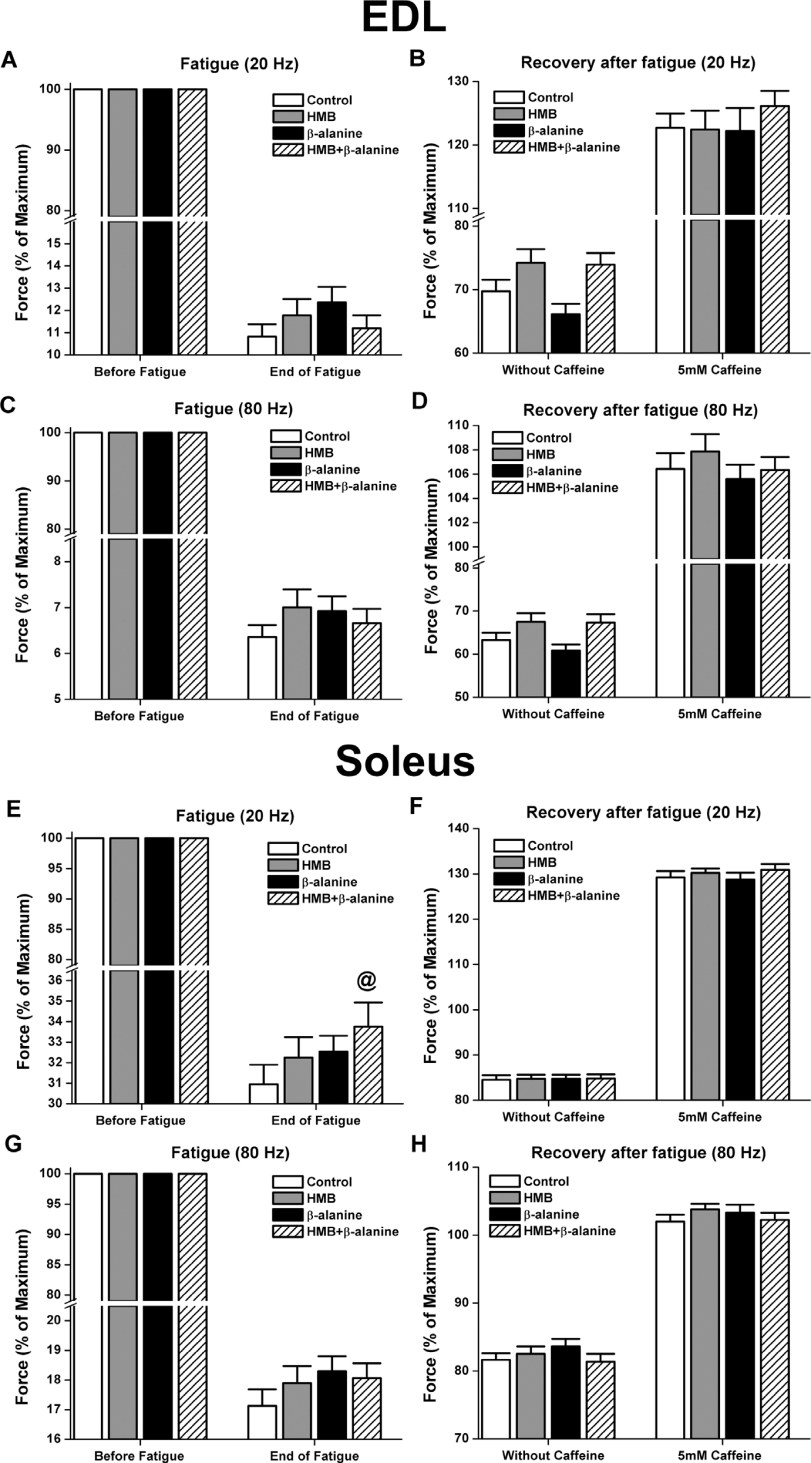
Fatiguing stimulation and recovery from fatigue in EDL and soleus muscles. Alternating frequencies of 80 Hz and 20 Hz were used to fatigue the muscles intermittently for 5 minutes, with a periodicity of one second. The periodicity, or interval between stimulations, was then extended to one minute to allow force recovery in the absence and presence of 5 mM caffeine. A and B) EDL fatigue and recovery, respectively, using the low stimulatory frequency of 20 Hz. D) EDL fatigue and recovery, respectively using the high frequency of 80 Hz. E and F) Soleus fatigue and recovery, respectively, using the low stimulatory frequency of 20 Hz (**@** denotes P = 0.049). G and H) Soleus fatigue and recovery, respectively, using the high frequency of 80 Hz. (EDL: Control-n = 22 muscles, HMB-n = 23 muscles, *β*-alanine-n = 24 muscles, HMB + *β*-alanine-n = 24 muscles. SOL: Control-n = 22 muscles, HMB-n = 24 muscles, *β*-alanine-n = 24 muscles, HMB + *β*-alanine-n = 22 muscles).

We also investigated fatigue resistance and recovery from fatiguing contractions in both EDL and soleus muscles. No changes were observed in the EDL muscle (Fig 6A–6D). Although not statistically significant, there was a trend for reduction in soleus muscle fatigue in the HMB and *β*-alanine groups, and especially the combination thereof (HMB + *β*-alanine: 5.1% increase in force after fatigue compared to force prior to fatigue, compared to control diet, P = 0.049) at the submaximal frequency of stimulation (20 Hz) when compared to control diet (Fig 6E).

## Discussion

In this study, we investigated the effects of both HMB treatment on cultured muscle cells and dietary HMB supplementation on muscle contractility in late middle-aged mice. In the latter study, we also tested for an interaction with *β*-alanine. A well characterized hallmark of aging skeletal muscle is the diminished capacity for regeneration of muscle after injury or exercise, and at least one contributing factor is the progressive loss of regeneration-associated satellite cells and myoblasts [76,77]. These resident mononuclear cells can be modeled in cell culture through study of isolated murine C_2_C_12_ myoblasts. We found that HMB treatment enhanced C_2_C_12_ myoblast proliferation (Fig 1C). In addition, HMB treatment enhanced C_2_C_12_ myoblast viability, even under the unfavorable conditions of serum reduction (Fig 1A and 1B). Taken together, HMB’s beneficial effects on myoblast viability and proliferation in cell culture align with a role in vivo on improving the regenerative capacity of myofibers throughout aging, as noted in a preclinical study of muscle unloading in a rat model [37]. Additionally, we provide compelling evidence that HMB directly enhances SR calcium release and/or storage in muscle cells (Fig 2A–2C). HMB treatment increased the KCl-elicited calcium response in fully differentiated C_2_C_12_ myotubes, suggesting that overall depolarization-coupled calcium release was enhanced. HMB also increased the caffeine-elicited response, suggesting that the calcium-induced calcium release (CICR) properties were enhanced. The larger ionomycin response further suggests that HMB increased total Ca^+2^ storage within the SR of C_2_C_12_ myotubes [68,78]. In our Ca^+2^ imaging system, a ΔFura-2 ratio change of 0.1 units is equivalent to approximately a [Ca^+2^]_i_ of 125 nM [79]. Thus, HMB induced an increase of 400–500 nM more Ca^+2^ release from the SR. Another important observation is that HMB appears to spare the cells from the natural decline in calcium release due to the order effect of consecutive chemical stimulations (Fig 2C), suggesting that these cells are more intact, or that E-C coupling is preserved. The absence of an effect of 25 μM HMB treatment on KCl and caffeine-induced calcium release in myotubes may suggest a sufficient dose requirement for HMB to modulate SR calcium release in muscle cells. To our knowledge, this is the first ever report of the beneficial effect of HMB treatment on calcium handling and EC-coupling in muscle cells.

In the late middle-aged mouse contractility study, a key observation is that the dietary HMB and *β*-alanine interventions generally affected muscle strength and quality to a greater extent in the EDL muscle than the soleus. It is well established that the detrimental effects of aging occur to a greater extent and at an earlier age at onset within muscle groups predominantly comprised of fast twitch myofibers, such as the EDL muscle, while slow twitch myofiber-predominant muscles, like the soleus muscle, have a tendency to resist atrophy before late old age [80,81]. Both the absolute twitch force and maximal tetanic force were increased in the EDL muscles of mice supplemented with *β*-alanine (Fig 4A). *β*-alanine also increased the rate of force development in EDL at all stimulatory frequencies (Fig 5A). These data suggest that *β*-alanine supplementation improved the biomechanical properties of fast twitch myofibers found at high frequency in the EDL muscle. We also observed an increase in absolute force and muscle quality (force normalized to muscle cross sectional area) at the lower frequency of stimulation (20 Hz) in EDL from mice co-supplemented with HMB and *β*-alanine. Additionally, the soleus muscles from the same group exhibited a trend for enhanced resistance to fatiguing stimulations at 20 Hz, which was not observed in the EDL muscle. Enhanced muscle contractility at the frequencies of 1 Hz and 20 Hz is very physiologically relevant, especially when considering that mammalian skeletal muscles normally function in this range [82,83]. Improved contractile force in fast twitch myofibers as well as enhanced fatigue resistance in slow twitch myofibers may confer an additive advantage for sustaining skeletal muscle strength and stamina for mobility.

It is interesting that neither muscle mass nor normalized force in EDL were different for *β*-alanine at the maximal stimulation frequencies, yet so for absolute force. It is possible that the slight relative increase in EDL muscle wet weight observed with *β*-alanine supplementation, combined with the slight relative increase in normalized force, interacted to generate the statistically significant absolute force change compared to control mice. It is unclear from these slight changes whether muscle mass or improved contractility were the main driver for increased absolute force. Regardless of muscle quality, absolute force likely reflects the more important translational outcome in humans, at least under conditions where hypertrophic signals can be activated. Such is the case for many experimental therapies targeting myostatin blockade, in which case muscle strength is enhanced without improvement of muscle quality [84]. From a pure force per muscle weight perspective, normalized force data better describe the contractile physiology of individual myofibers than absolute force data. However, absolute force data, we believe, are more relevant to whole muscle strength and thus quality of life for an aging human, for which these studies attempted to model. It is after all, muscle’s absolute force that facilitates our daily activities. Absolute force data are also important because fibrosis and intramuscular fat accumulation can muddle interpretation of normalized forced data, at least when normalized to whole muscle wet weight and even CSA. An alternate analysis is to normalize to myofibrillar protein content, however we did not collect such lysates in this study.

We detected an increase in EDL muscle strength in all experimental groups except for the HMB group, while the soleus muscles supplemented with all experimental diets did not show any force benefit. On the other hand, HMB supplementation did have an effect on the time it took to reach peak contractile force in both EDL and soleus muscles (Fig 5B and 5E), suggesting enhanced excitation-contraction coupling. We speculate that such an outcome may provide benefit in a high coordination task such as the rotarod performance test, and this will be the subject of future study. It is interesting that the effects of HMB in late middle-aged, 19 month old pre-sarcopenic mice manifest primarily in the kinetic properties of force development, while the magnitude of force production is unaffected. It is possible that the benefits of HMB supplementation on the magnitude of muscle force would have been more pronounced in a mouse model at or greater than 24 months of age, when sarcopenia is predominant and significant muscle atrophy has occurred. Other reasons for the lack of HMB effect on muscle force in this study may pertain to the modest dose (equivalent to 3 g/day in humans) and duration of supplementation, designed to mimic what could be tested in a human clinical study. Therefore, additional studies in older mice with additional doses and perhaps more translational in vivo phenotyping are warranted.

It is also important to note that the effects of the combination of HMB and *β*-alanine on force generation in the EDL muscle and fatigue resistance in the soleus at the submaximal frequency of stimulation of 20 Hz are greater than the effects of either ingredient alone. It is unclear exactly how these small molecules interact to produce these complementary effects in muscle. It has been shown that *β*-alanine alone can increase calcium sensitivity of contractile proteins [57,58]. Force generation at 20 Hz is heavily influenced by alterations in E-C coupling and especially calcium release from the sarcoplasmic reticulum [71]. Given that we observed improved calcium handling upon HMB treatment of cultured myotubes, we speculate that HMB and *β*-alanine may act to both improve calcium handling. This effect of HMB, however, needs to be tested in vivo, and likewise, it will be important to test *β*-alanine’s effect on calcium release in our myoblast model.

In summary, we found that *β*-alanine increased both the 1 Hz and 80 Hz-stimulated absolute forces of EDL muscles in late middle-aged mice. Of utmost importance, even producing more force, these muscles were not prone to damage, even when subjected to our arduous fatiguing stimulation protocol. *β*-alanine supplementation also improved the rate of force generation at all tested frequencies. When dietary *β*-alanine was combined with HMB, the increase in EDL force occurred at physiologically relevant 20 Hz frequency for absolute and normalized force. In soleus muscle, the effects of each of HMB, *β*-alanine, and the combination thereof reduced time to reach peak force. Altogether, these data support the concept that dietary supplementation with HMB and *β*-alanine might help counteract the decline in muscle function during aging, and preserve muscle function during repetitive bouts of activity. We also report a novel mechanism of action for HMB in enhancing calcium release from the SR, suggesting that HMB improves E-C coupling in muscle cells.

## Supporting Information

S1 ARRIVE Checklist ARRIVE Guidelines Checklist. (DOCX)
