# Supplementary material for: Cellular and Physiological Effects of Dietary Supplementation with β-Hydroxy-β-Methylbutyrate (HMB) and β-Alanine in Late Middle-Aged Mice
Source: PLoS One. 2016 Mar 8;11(3):e0150066. doi: 10.1371/journal.pone.0150066 (PMC4783107; doi:10.1371/journal.pone.0150066)

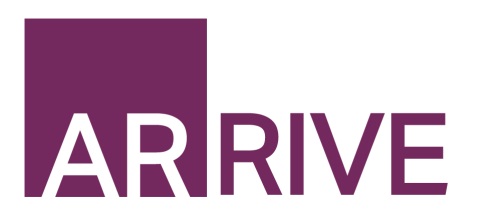


The ARRIVE Guidelines Checklist

Animal Research: Reporting In Vivo Experiments

Carol Kilkenny^1^, William J Browne^2^, Innes C Cuthill^3^, Michael Emerson^4^ and Douglas G Altman^5^

*^1^The National Centre for the Replacement, Refinement and Reduction of Animals in Research, London, UK, ^2^School of Veterinary Science, University of Bristol, Bristol, UK, ^3^School of Biological Sciences, University of Bristol, Bristol, UK, ^4^National Heart and Lung Institute, Imperial College London, UK, ^5^Centre for Statistics in Medicine, University of Oxford, Oxford, UK.*

|  | | ITEM | RECOMMENDATION | Section/ Paragraph |
| --- | --- | --- | --- | --- |
| 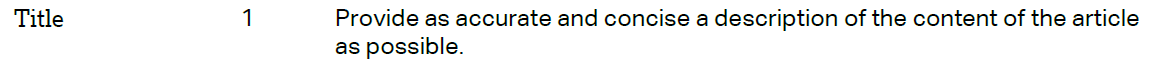 | | | p.1 |  |
| 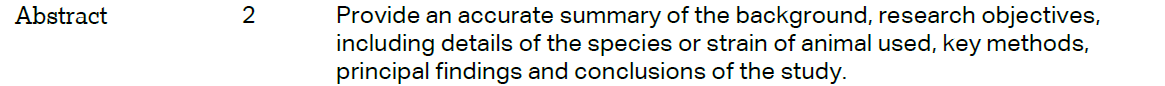 | | | p. 2 |  |
| INTRODUCTION | | |  |  |
| 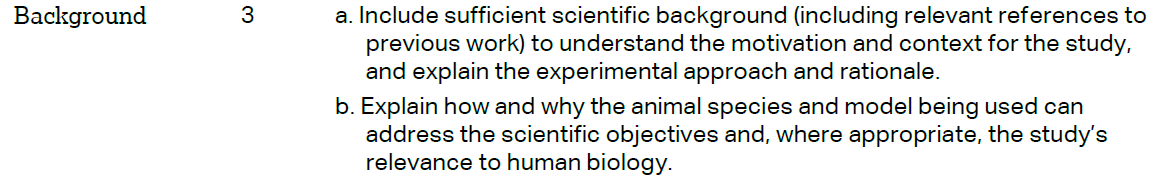 | | | p. 3-5 |  |
| 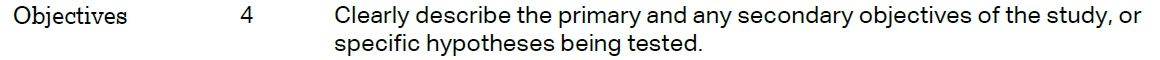 | | | p. 5 |  |
| METHODS | | |  |  |
| 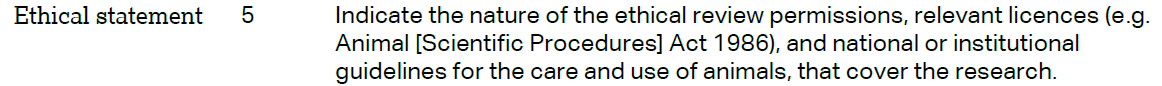 | | | p. 7 |  |
| 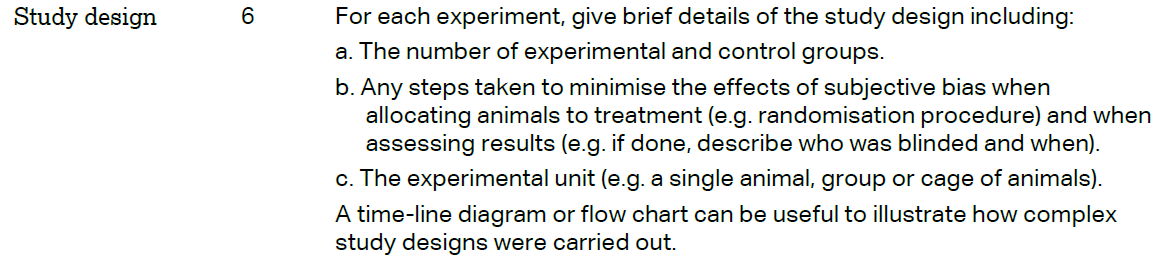 | | | p. 7-8 |  |
| 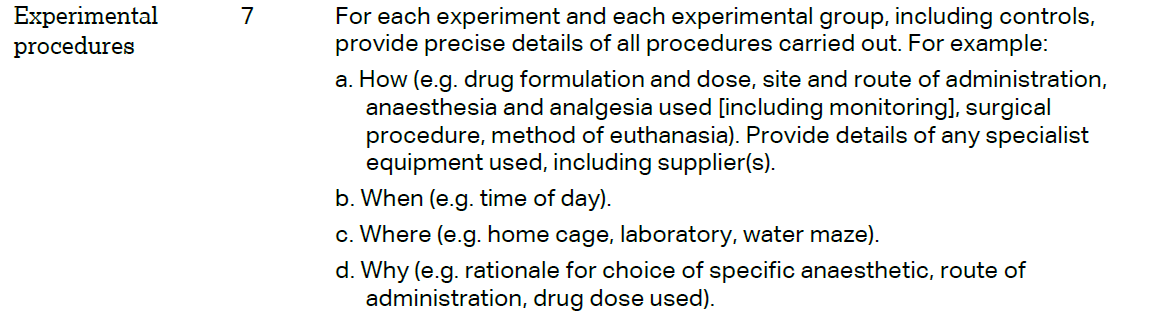 | | | p. 7-10 |  |
| 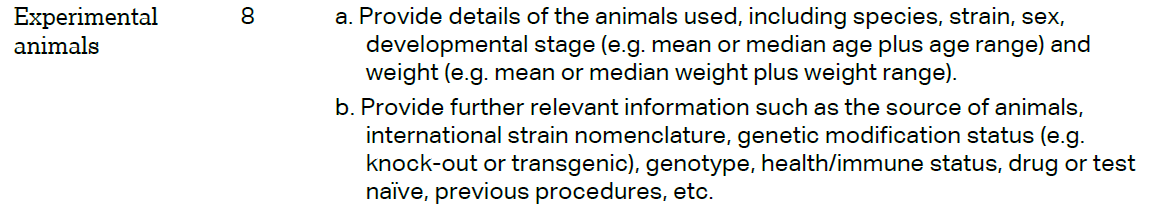 | | | p. 7-8 |  |

The ARRIVE guidelines. Originally published in *PLoS Biology*, June 2010^1^

| 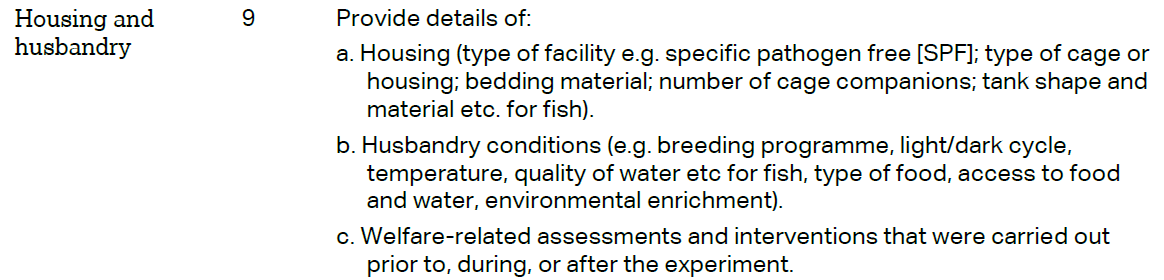 | p. 7-8 | |
| --- | --- | --- |
| 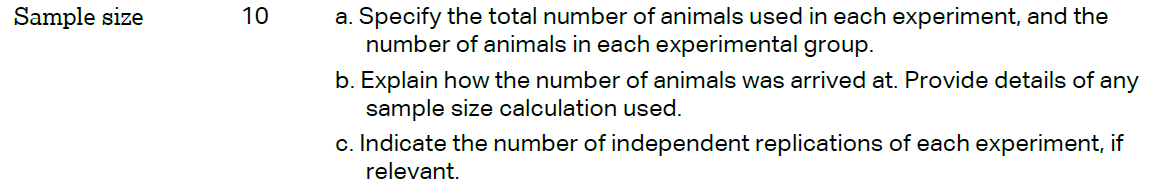 | p. 7-8 | |
| 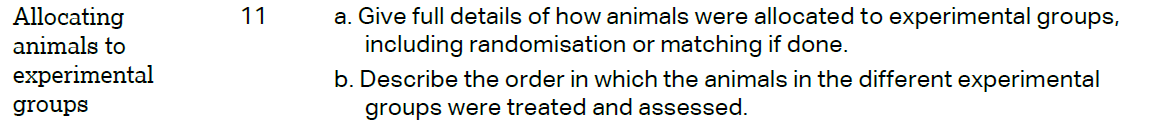 | p. 7-8 | |
| 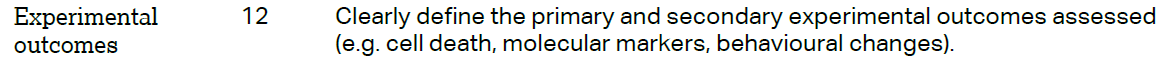 | p. 8-10 | |
| 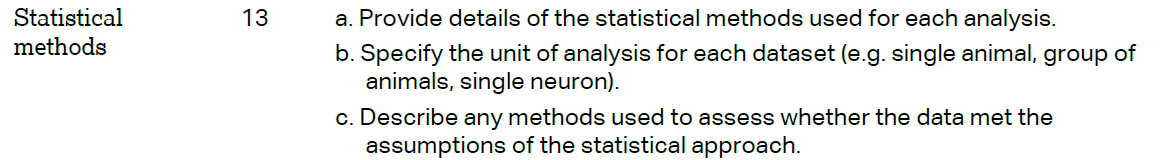 | p. 10-11 | |
| RESULTS |  | |
| 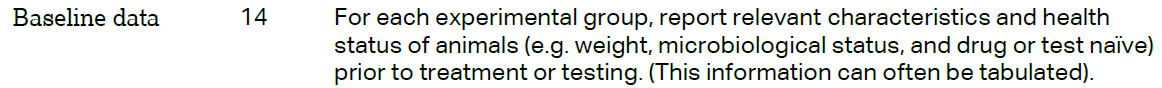 | p. 13-14 | |
| 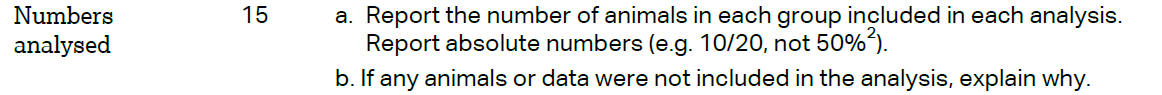 | p. 13-17 | |
| 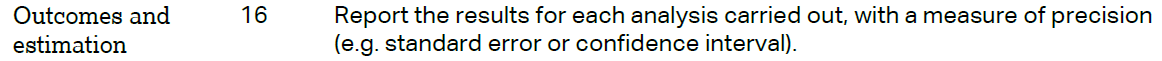 | p. 13-17 | |
| 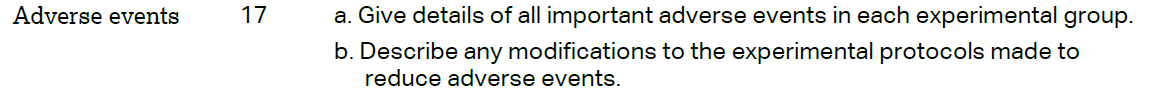 | p. 13-17 | |
| DISCUSSION |  | |
| 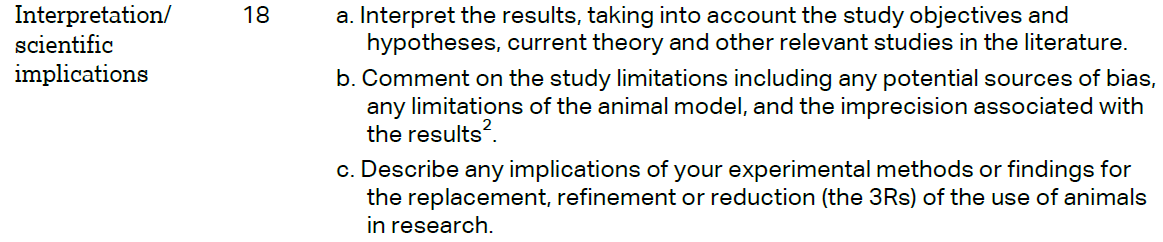 | p. 18-21 | |
| 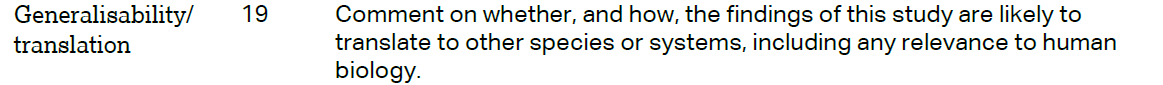 | p. 18-21 | |
| 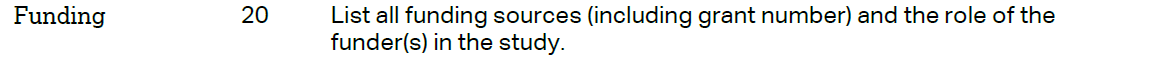 | |  |


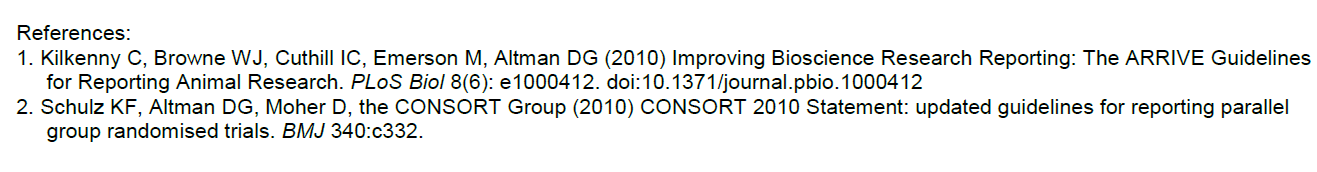

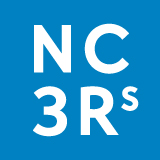

Supplement: S1 ARRIVE Checklist — (DOCX) [file pone.0150066.s001.docx]
